# Supplementary material for: CSF1R Inhibition Combined with GM-CSF Reprograms Macrophages and Disrupts Protumoral Interplays with AML Cells
Source: Cancers (Basel). 2021 Oct 21;13(21):5289. doi: 10.3390/cancers13215289 (PMC8582394; doi:10.3390/cancers13215289)
Supplement: Supplementary file 1 [file cancers-13-05289-s001.zip › Supp. fig. with legends.pdf]

Article

CSF1R Inhibition Combined with GM-CSF Reprograms Macrophages and Disrupts Protumoral Interplays with AML Cells

Tatiana Smirnova *,†, Caroline Spertini † and Olivier Spertini *

Supplementary Materials

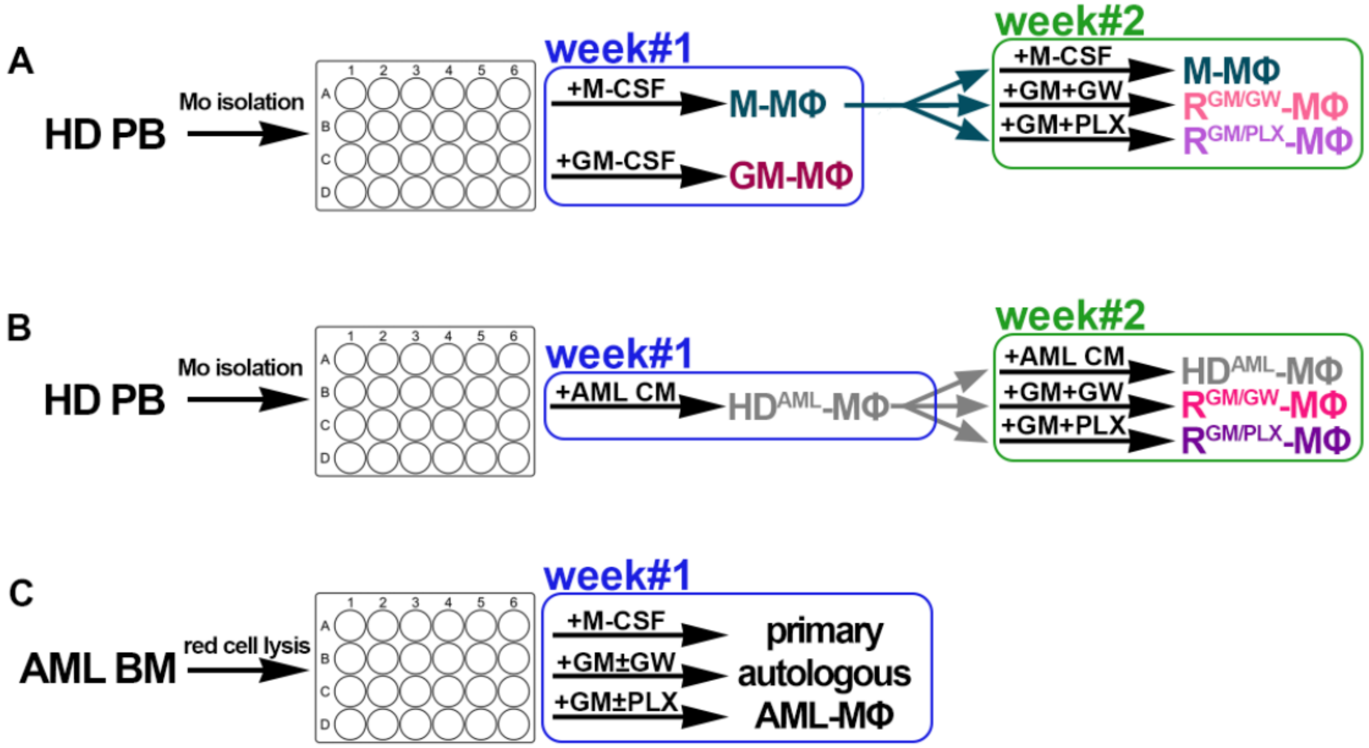

Figure S1. Schematic protocol of MΦ differentiation process. (**A**) M- and GM-MΦs were obtained from HD PB monocytes after one week of culture in PM supplemented as indicated. They were characterized in Figure 1B (and S3, S4A,C) and used for co-cultures experiments in Figures 2C,D and 5A,D; their CM was analyzed in Figures 6, 7 and 9B (and S5C). After an additional week of culture of M-MΦ with indicated inhibitors and/or supplements, we obtained M-, $R^{GM/GW}$- and R-$^{GM/PLX}$-MΦs, that were characterized in Figure 3A (and S6A, S7A) and used in co-cultures of Figures 3B-D and 5B,C,E. CM of $R^{GM/GW}$-MΦs was used in Figures 6 and 7 (and S5C). (**B**) HD^{AML}-MΦs were differentiated from HD PB monocytes cultured in PM supplemented with myeloblast CM for one week and characterized in Figure 1C,D (and S4B,D). They were further differentiated for another week as indicated, analyzed in Figure 4A (and S6B) and used in co-culture experiments in Figure 4B,C. (**C**) Primary autologous AML-MΦs from BM patient samples were analyzed at day 0 (Figure 1A), co-cultured on HD monocytes in Figure 2A,B, or cultured for one week after red cell lysis in medium supplemented as indicated. Their phenotype was analyzed in Figure 4D (and S6C,D, S7B-D) and their CM in Figures 1E and 8 (and S5A,B,D).

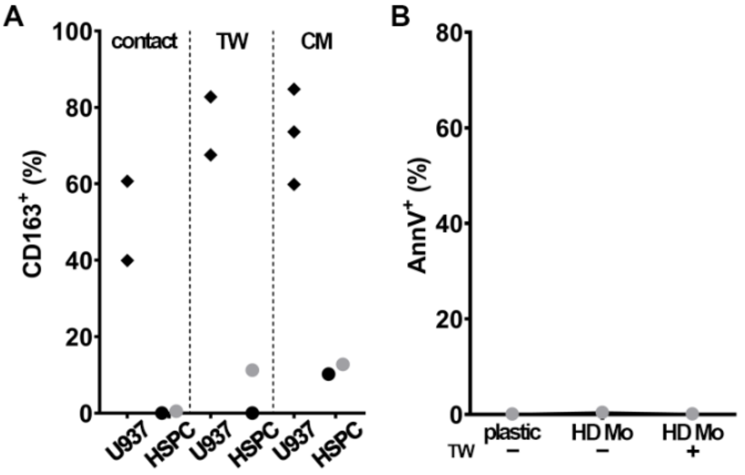

Figure S2. HSPC do not upregulate CD163 expression on MΦs. (**A**) HSPC were obtained by erythrocyte lysis from normal BM (black dots) and PB leukapheresis enriched in CD34⁺ cells by HSPC mobilization (gray dots). They were co-cultured for 3 days with HD monocytes in direct contact (contact), or in 0.4 µm transwell inserts (TW), or with their conditioned medium (CM) and their impact on MΦ CD163 expression was compared to that of U937 (black diamonds). (**B**) HSPC from PB leukapheresis were co-cultured for 3 days as indicated (plastic vs. monocytes +/− TW) to monitor their survival.

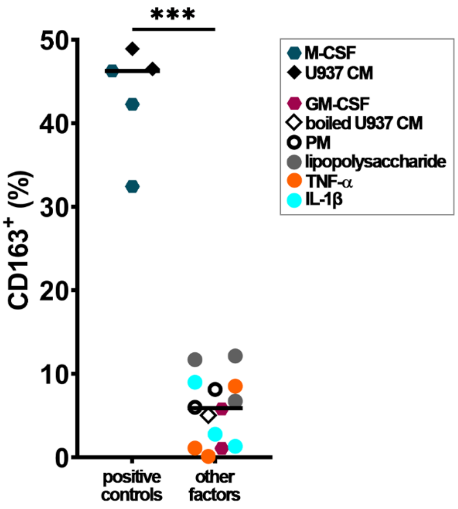

Figure S3. Inflammatory or inactivated factors do not upregulate CD163 expression on HD MΦs. Monocytes from 1-3 HD were cultured with indicated activating (positive controls) or non-activating (other factors) conditions for 7 days, after which CD163 expression was analyzed by FC. *** $p < 0.001$.

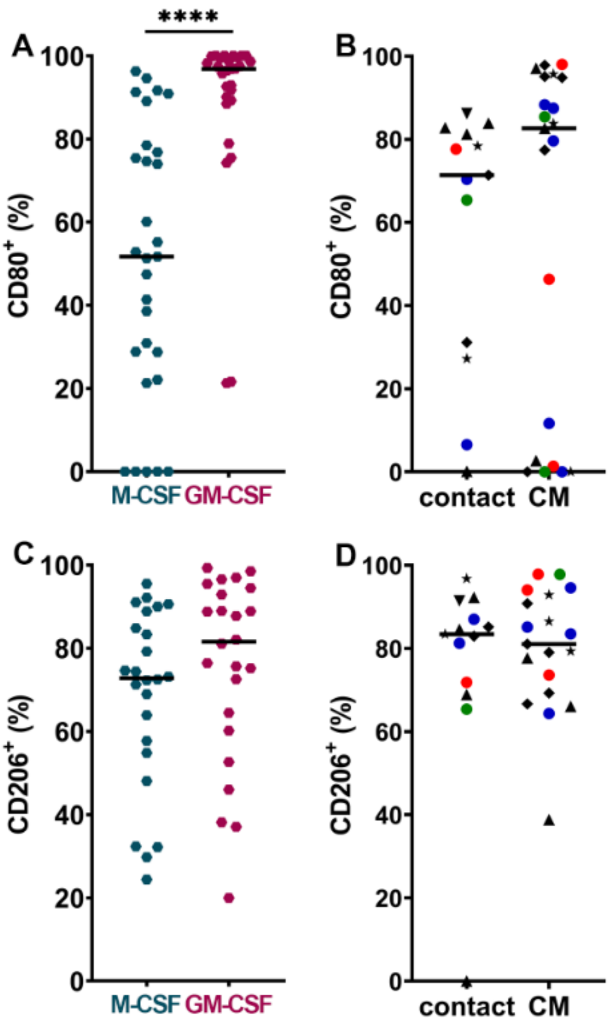

Figure S4. CD80 and CD206 are frequently expressed by HD and HD^{AML}-MΦs. (**A**) Expression frequency of CD80 in HD MΦs differentiated in PM supplemented with M- or GM-CSF. n = 28-29 **** $p < 0.0001$. (**B**) CD80 expression frequency in allogeneic HD^{AML}-MΦs obtained from HD Mo cultured with AML cell lines or primary patient blasts (contact) or CM. n = 13-21. (**C**) Expression frequency of CD206 in HD MΦs that have been cultured in M- or GM-CSF for one week. n = 24. (**D**) CD206 frequency of expression in allogeneic HD^{AML}-MΦs that have been cultured with AML cell lines or primary patient blasts (contact) or CM for one week. n = 13-19. In all four panels, the median is indicated by a black horizontal line. Symbols in (B) and (D) are as follows: ★ = HL-60, ▲ = NB4, ◆ = U937, ▼ = OCI-AML3, and dots = primary patient blasts color-coded according to genetic risk (green = favorable, blue = intermediate and red = high risk).

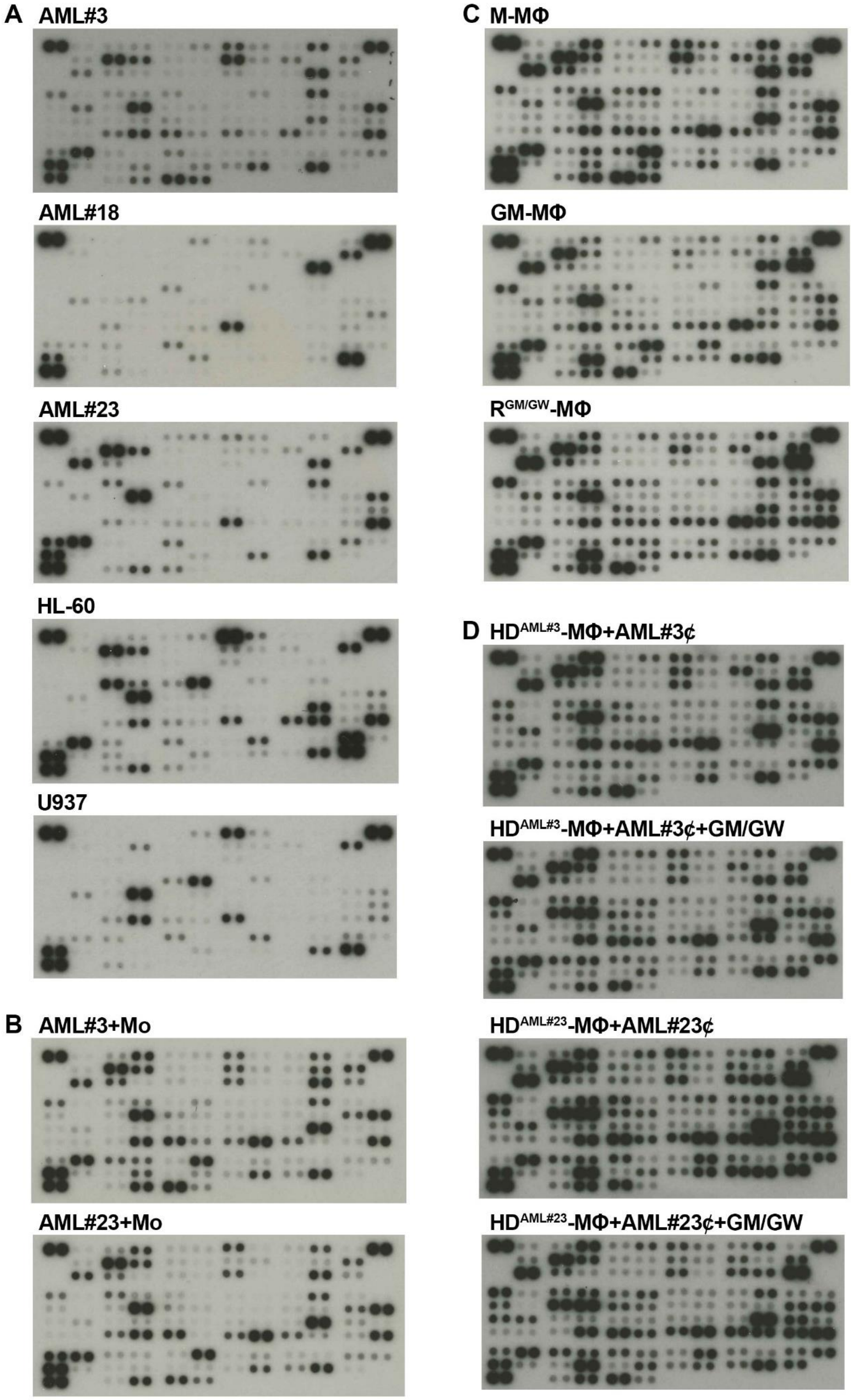

Figure S5. Autoradiographies of cytokine array membranes used for densitometry; origin of CM is indicated above each membrane. Membranes were quantified and illustrated in (**A**) Figure 1E and 2B, (**B**) Figure 2B, (**C**) Figure 7 and (**D**) Figure 8. ¢ = cells.

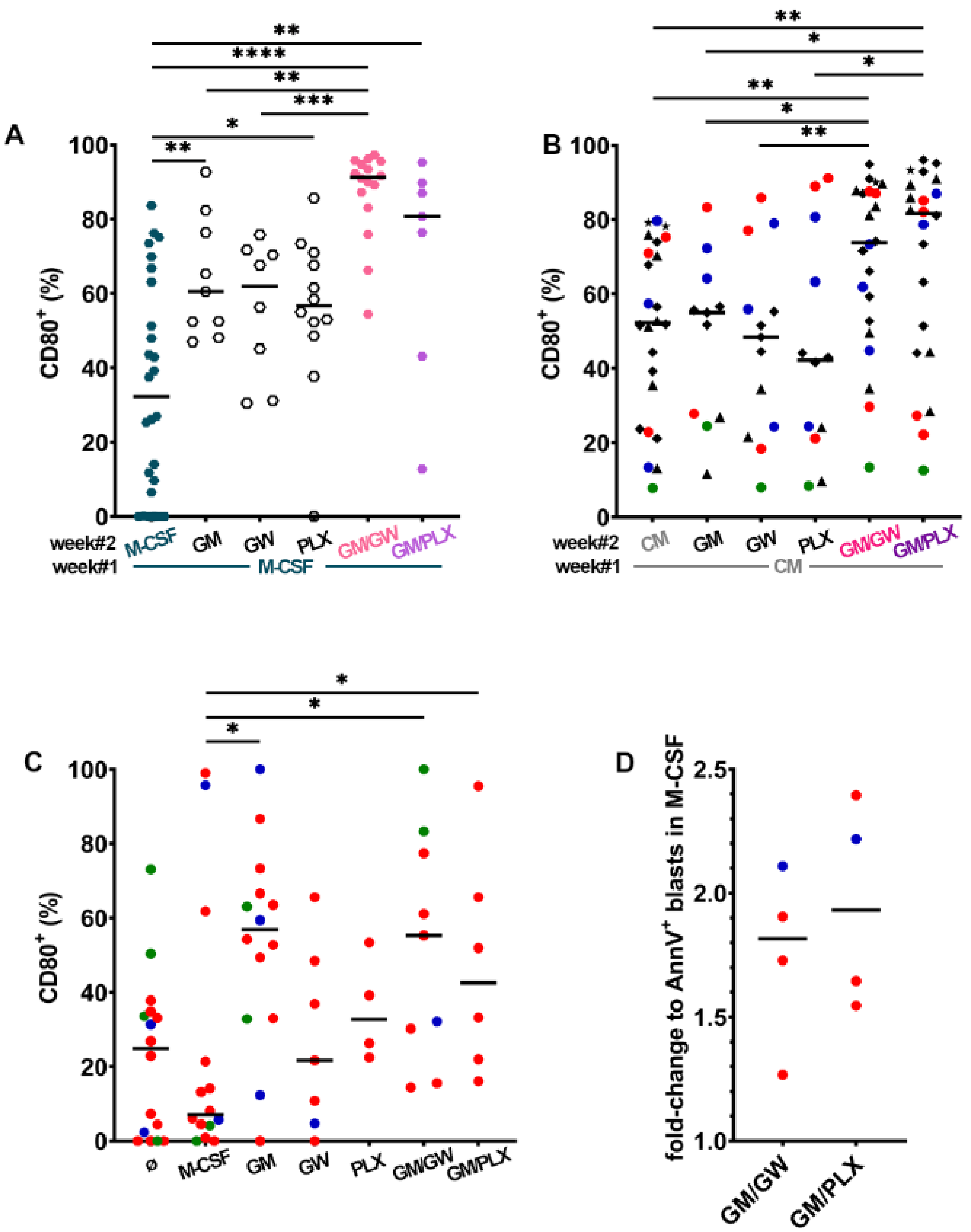

Figure S6. CD80 expression is increased in R-MΦs. (**A**) CD80 expression frequency in HD MΦs after two weeks of culture in PM with indicated supplements. n = 7-26. (**B**) CD80 expression frequency in allogeneic HD^{AML}-MΦs after two weeks of culture in PM supplemented with CM or indicated inhibitors. Symbols indicate origin of CM as follows: ★ = HL-60, ▲ = NB4, ◆ = U937, and dots = primary patient blasts color-coded according to genetic risk. n = 11-24. (**C**) CD80 expression frequency on MΦs from autologous BM co-cultured for one week in PM with indicated supplements. n = 4-16. (**D**) AnnV positivity of primary patient blasts was analyzed in AML BM co-cultures. Apoptosis of blasts cultured in PM supplemented with GM/GW or GM/PLX was normalized to apoptosis measured on blasts co-cultured in PM supplemented with M-CSF. Blasts are color-coded according to genetic risk and are from patients #16, #17, #26, #23, and #34. In all panels, GM = GM-CSF, GW = GW2580, PLX = PLX3397, GM/GW = GM-CSF + GW2580, GM/PLX = GM-CSF + PLX3397. Green = favorable, blue = intermediate and red = high risk. * $p < 0.05$, ** $p < 0.01$, *** $p < 0.001$, **** $p < 0.0001$.

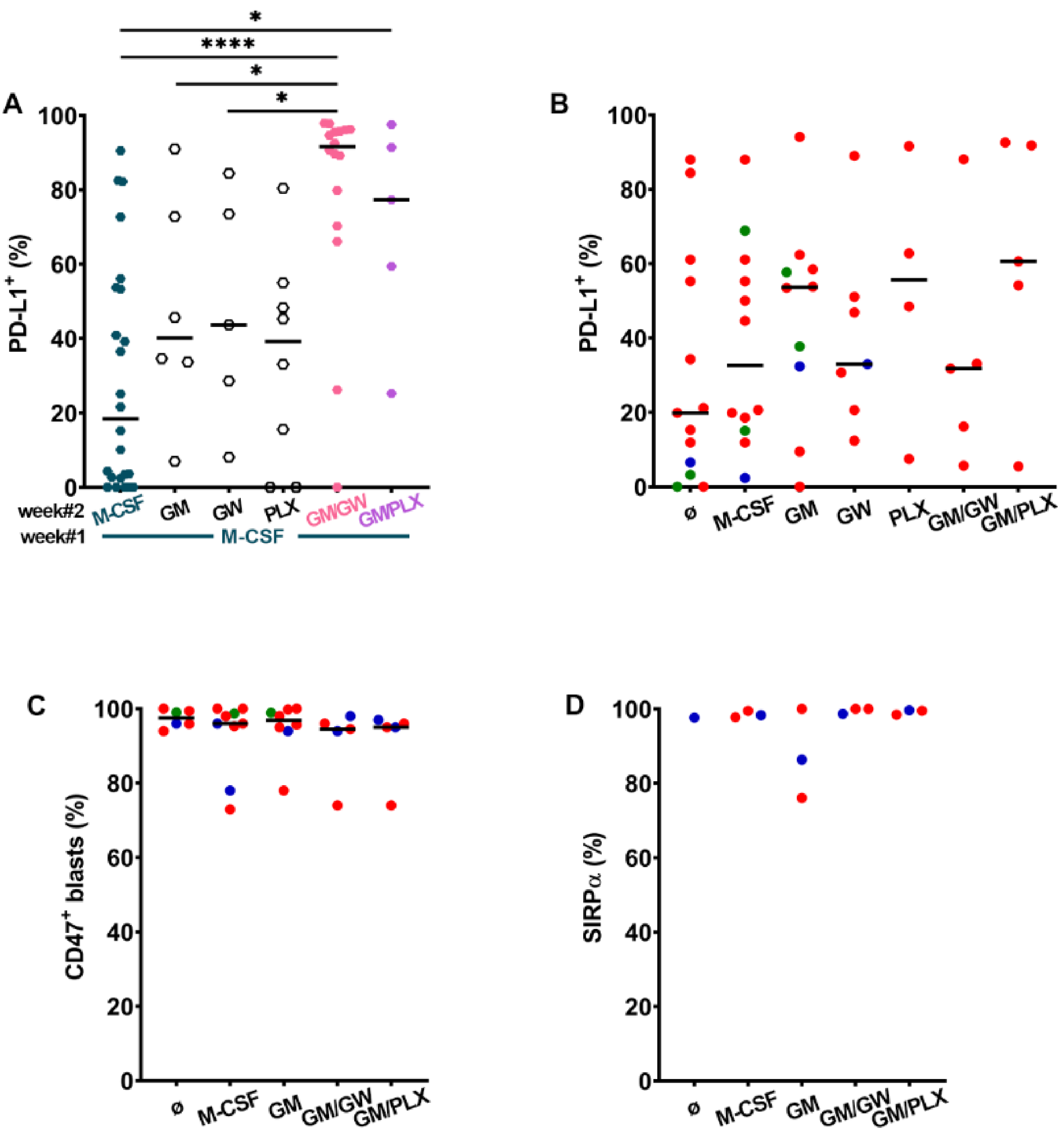

Figure S7. PD-L1 is increased on R-MΦs, while SIRP*α* is unchanged. (**A**) PD-L1 expression frequency in HD MΦs after two weeks of culture in PM with indicated supplements. n = 5-24. * $p < 0.05$ and **** $p < 0.0001$. (**B**) Frequency of PD-L1 expression on MΦs from autologous BM co-cultured for one week in PM with indicated supplements. n = 4-13. (**C**) Expression frequency of CD47 on blasts from autologous BM co-cultures after one week. n = 5-9. (**D**) Frequency of expression of SIRP*α* on MΦs from autologous BM co-cultures. n = 1-3.

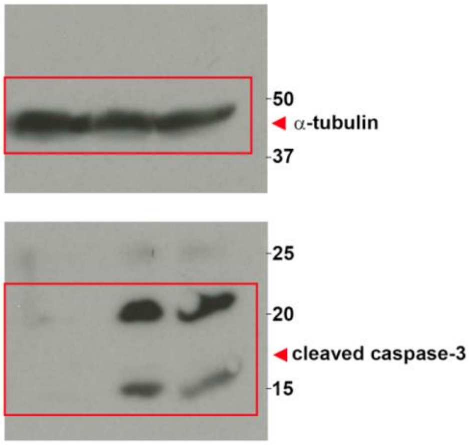

Figure S8. Uncropped western blot of Figure 6C.
