# Supplementary material for: CSF1R Inhibition Combined with GM-CSF Reprograms Macrophages and Disrupts Protumoral Interplays with AML Cells
Source: Cancers (Basel). 2021 Oct 21;13(21):5289. doi: 10.3390/cancers13215289 (PMC8582394; doi:10.3390/cancers13215289)
Supplement: Supplementary file 1 [file cancers-13-05289-s001.zip › Supp. tables.pdf]

Table S1: FAB, WHO 2016 and ELN 2017 genetic risk classifications from included patients.
t-AML: therapy-related AML, MRC: myelodysplasia-related changes, RGA: recurrent genetic abnormalities.

patient#	FAB	WHO 2016	ELN 2017 genetic risk	Samples
1	AML M1	AML with RGA	Favorable	PB
2	AML M4Eo	AML with RGA	Favorable	BM & PB
3	AML M5	AML with RGA	Favorable	BM & PB
4	AML M4	AML with RGA	Favorable	BM & PB
5	AML M4	AML with RGA	Favorable	BM & PB
6	AML M2	AML with RGA	Favorable	BM
7	AML M2	AML with RGA	Favorable	BM
8	AML M4	AML with RGA	Favorable	BM & PB
9	AML M2	AML with RGA	Favorable	PB
10	AML M2	AML with maturation	Intermediate	BM
11	AML M2	AML with RGA	Intermediate	BM
12	AML M5	AML with RGA	Intermediate	BM
13	AML M2	AML with RGA	Intermediate	PB
14	AML M2	AML with RGA	Intermediate	BM & PB
15	AML M2	AML with RGA	Intermediate	PB
16	AML M4	AML with RGA	Intermediate	PB
17	AML M2	AML with RGA	Intermediate	BM & PB
18	AML M4	AML with RGA	Intermediate	PB
19	AML M5	AML with RGA	Adverse	BM
20	AML M5	AML with MRC	Adverse	PB
21	AML M4	Monoblastic AML	Adverse	BM
22	AML M5	Monoblastic AML	Adverse	BM
23	AML M4	AML with MRC	Adverse	BM & PB
24	AML M2	AML with MRC	Adverse	BM
25	AML M2	AML with maturation	Adverse	BM
26	AML M1	AML without maturation	Adverse	BM
27	AML M4	AML with MRC	Adverse	PB
28	AML M1	AML with MRC	Adverse	BM
29	AML M4	AML with MRC	Adverse	BM
30	AML M5	AML with MRC	Adverse	BM
31	AML M1	AML with MRC	Adverse	BM & PB
32	AML M4	AML with MRC	Adverse	PB
33	AML M1	AML with MRC	Adverse	BM
34	AML M4	AML with MRC	Adverse	BM
35	AML M2	AML with maturation	Adverse	BM & PB
36	AML M4	AML with MRC	Adverse	BM
37	AML M4	Myelomonocytic AML	Adverse	BM
38	AML M5	t-AML	Adverse	BM
39	AML M2	AML with maturation	Adverse	BM
40	AML M4	t-AML	Adverse	BM
41	AML M2	AML with maturation	Adverse	BM
42	AML M2	AML with maturation	Adverse	PB

Table S2: Antibodies for FC.

Target (human)/clone	Fluorophore	Product #	Supplier
CD45	KrO	B36294	Beckman Coulter
CD14 clone TUK4	PE	R0864	Dako
CD14 clone 63D3	APC	367118	Biolegend
CD163 clone GHI/61	BV 421	333612	Biolegend
CD80 clone 2D10	APC	305220	Biolegend
CD206 clone 15-2	FITC	321104	Biolegend
SIRPα/β clone SE5A5	PE/Cy7	323808	Biolegend
CD47 clone CC2C6	Alexa Fluor 700	323126	Biolegend
PD-L1 clone MIH1	PE-Cyanine 7	25-5983-42	eBioscience
CSF-1R clone 9-4D21E4	PC5.5	347 310	Biolegend
CD34	Alexa Fluor 700 or 750	A86354 or B92463	Beckman Coulter
CD38	ECD	A99022	Beckman Coulter
CD117	PC5.5	B96754	Beckman Coulter
CD16	PC7	6607118	Beckman Coulter
CD4	PC7	737660	Beckman Coulter
CD8	PC7	737661	Beckman Coulter
CD3	APC-Alexa Fluor 750	A94680	Beckman Coulter
CD56	PC5.5	B49189	Beckman Coulter
CD123	PC7	B13647	Beckman Coulter
CD19	APC-Alexa Fluor 700	B49212	Beckman Coulter

Table S3: Relative quantification of all cytokines from membranes depicted on Figure S5.

	AML#3	AML#18	AML#23	HL-60	U937	AML#3 +Mo	AML#23 +Mo	M-MΦ	GM-MΦ	R$^{GM/GW}$-MΦ	HD$^{AML\#3}$-MΦ	HD$^{AML\#3}$-MΦ +GM/GW	HD$^{AML\#23}$-MΦ	HD$^{AML\#23}$-MΦ +GM/GW
**adiponectin**	ND	ND	ND	ND	ND	ND	ND	126.2	ND	122.1	59.2	107.0	48.2	79.3
**Ang-1**	ND	ND	45.8	ND	ND	46.1	66.5	83.0	37.0	51.5	54.0	87.3	109.9	85.6
**Ang-2**	134.3	71.5	67.5	57.6	38.5	74.3	59.9	271.0	215.3	260.7	210.7	368.5	240.6	290.0
**angiogenin**	109.0	ND	ND	ND	ND	749.4	558.0	714.7	295.7	511.3	953.3	829.1	977.0	861.7
**ApoA1**	ND	ND	ND	ND	ND	177.2	200.9	151.2	109.0	119.2	137.4	189.9	80.3	115.4
**BAFF**	510.8	ND	108.7	1108.6	661.3	411.8	553.9	536.2	85.2	187.3	392.5	638.6	402.2	314.8
**Basigin**	362.4	417.0	225.6	591.9	331.7	573.8	425.8	737.3	581.0	768.0	438.6	629.5	448.5	488.1
**BDNF**	128.6	50.8	47.6	203.2	ND	ND	ND	312.2	204.3	224.7	127.3	148.1	94.2	124.6
**C5/C5a**	ND	ND	ND	ND	ND	ND	66.1	70.8	ND	54.2	70.1	160.4	72.7	121.1
**CCL17**	211.6	ND	ND	ND	ND	466.3	74.8	380.2	960.2	995.2	116.0	966.0	121.1	956.0
**CCL19**	108.4	37.0	ND	50.6	22.4	61.1	124.9	141.5	164.2	727.0	56.2	902.4	109.5	839.2
**CCL2**	753.0	ND	ND	375.3	526.3	833.5	801.3	701.1	677.7	678.6	846.6	845.5	767.6	762.7
**CCL20**	104.8	ND	ND	644.1	ND	122.4	186.4	200.3	381.3	790.3	129.8	1006.1	609.6	714.2
**CCL3/4**	404.4	ND	ND	347.2	ND	421.8	384.2	420.9	860.1	936.2	66.4	844.8	155.8	736.0
**CCL5**	71.8	ND	ND	236.4	140.9	148.3	148.4	170.8	567.2	257.9	175.2	628.7	168.9	611.3
**CCL7**	470.7	ND	42.4	70.8	37.6	739.4	719.5	520.9	297.3	444.3	541.5	909.4	726.1	763.9
**CD14**	571.9	ND	43.2	ND	ND	459.5	427.4	683.0	367.5	499.3	490.0	499.8	333.2	407.0
**CD26**	247.3	18.7	50.2	24.7	ND	60.7	ND	459.7	178.3	439.7	559.3	306.9	355.3	245.4
**CD30**	ND	48.8	ND	ND	ND	ND	ND	110.0	83.6	129.7	43.6	130.3	42.5	65.7
**CD40L**	83.5	ND	ND	ND	ND	59.9	79.4	176.7	100.1	226.4	99.7	188.3	93.1	135.7
**CD71**	201.5	83.0	ND	53.4	ND	218.9	94.2	487.4	324.6	413.9	232.1	228.5	256.9	353.4
**CFD**	563.1	ND	396.1	432.2	103.2	691.8	602.4	666.7	326.3	625.4	684.9	716.6	633.6	560.1
**CHI3L1**	939.3	ND	881.7	850.4	ND	999.1	982.0	975.8	935.8	965.0	990.2	1026.6	968.7	982.8
**Cripto-1**	ND	ND	ND	ND	ND	ND	ND	ND	ND	ND	42.0	98.0	39.0	50.6
**CRP**	ND	ND	ND	ND	ND	44.9	64.0	86.5	50.6	93.7	71.7	181.3	65.0	66.6
**CXCL1**	242.5	ND	58.6	ND	ND	343.4	291.7	427.6	540.3	780.7	260.1	731.1	659.0	669.8
**CXCL10**	386.4	ND	ND	631.5	ND	997.4	994.9	920.3	265.4	659.3	1092.7	1134.2	1094.1	1113.8
**CXCL11**	64.1	ND	ND	ND	ND	ND	154.2	134.6	82.3	236.8	101.8	529.4	110.4	637.8
**CXCL12**	197.2	22.9	ND	48.3	ND	180.1	187.8	251.7	155.3	289.4	567.6	604.4	616.3	602.3
**CXCL4**	55.9	ND	ND	ND	ND	866.8	856.2	914.4	863.3	304.6	55.7	59.0	155.9	183.4
**CXCL5**	280.8	ND	630.2	ND	ND	652.3	575.2	944.8	930.7	1036.7	919.3	1007.6	938.3	1000.3
**CXCL9**	93.8	ND	ND	ND	ND	937.9	942.3	906.2	409.7	480.3	962.2	961.6	944.2	886.3
**Cystatin C**	938.7	ND	ND	156.8	ND	450.1	105.4	839.8	239.6	531.3	441.6	192.8	263.1	183.2

	AML#3	AML#18	AML#23	HL-60	U937	AML#3 +Mo	AML#23 +Mo	M-MΦ	GM-MΦ	R$^{GM/GW}$-MΦ	HD$^{AML\#3}$-MΦ	HD$^{AML\#3}$-MΦ +GM/GW	HD$^{AML\#23}$-MΦ	HD$^{AML\#23}$-MΦ +GM/GW
**Dkk-1**	122.5	ND	ND	37.6	ND	90.3	89.9	158.7	98.0	112.5	88.3	99.7	55.1	80.7
**EGF**	ND	ND	ND	ND	ND	556.2	115.8	303.7	102.0	ND	76.0	120.9	53.7	65.9
**Endoglin**	152.3	ND	93.1	ND	ND	496.4	342.4	561.7	247.3	474.6	251.7	344.0	245.3	294.9
**FasL**	74.3	ND	ND	ND	ND	ND	ND	140.5	90.4	149.5	95.7	166.3	76.9	77.2
**FGF-19**	107.5	ND	ND	43.4	ND	403.0	415.8	176.3	113.5	161.7	420.8	546.8	384.5	422.5
**FGF-2**	ND	ND	ND	ND	ND	68.7	72.0	58.5	35.2	60.5	65.9	183.8	58.2	61.3
**FGF-7**	ND	ND	ND	ND	ND	ND	ND	ND	ND	ND	39.8	89.4	32.1	51.0
**Flt-3 L**	ND	ND	ND	ND	ND	ND	ND	60.9	ND	ND	31.7	53.6	25.4	31.0
**G-CSF**	ND	ND	ND	ND	ND	ND	ND	ND	ND	72.2	39.6	617.5	98.0	408.6
**GDF-15**	928.6	821.9	464.3	ND	ND	879.6	722.6	933.7	842.3	887.4	710.9	794.6	674.8	397.4
**GH**	ND	ND	ND	ND	ND	ND	ND	ND	ND	ND	28.2	65.2	31.1	45.1
**GM-CSF**	110.5	ND	ND	ND	ND	289.3	177.0	696.1	1015.1	1057.0	842.3	1136.3	680.4	1123.4
**HGF**	103.5	ND	165.0	669.2	ND	129.9	348.3	235.2	87.1	145.1	168.4	187.9	241.2	281.8
**ICAM-1**	122.7	ND	ND	182.5	ND	281.7	197.7	288.5	191.9	367.5	292.8	657.3	404.3	438.2
**IFN-γ**	117.9	75.4	95.6	74.3	80.3	ND	97.2	277.1	209.8	321.1	231.1	487.9	106.1	199.3
**IGFBP-2**	ND	ND	ND	806.6	622.5	174.5	ND	448.1	ND	211.0	45.2	96.2	241.3	357.0
**IGFBP-3**	ND	ND	ND	ND	ND	ND	ND	ND	ND	ND	99.0	171.5	96.8	84.5
**IL-1 R4**	ND	ND	ND	52.4	ND	ND	ND	ND	ND	ND	36.3	81.0	48.4	75.6
**IL-10**	ND	ND	ND	ND	ND	223.7	161.4	145.8	ND	77.3	222.6	369.3	309.8	335.3
**IL-11**	56.0	ND	ND	ND	ND	83.9	47.4	133.5	66.5	105.5	161.4	258.6	169.5	184.5
**IL-12 p70**	ND	ND	ND	ND	ND	61.4	ND	64.1	ND	56.4	69.1	82.1	42.3	50.4
**Il-13**	ND	ND	ND	ND	ND	ND	ND	ND	ND	ND	53.1	95.8	46.2	69.1
**IL-15**	ND	ND	ND	ND	ND	52.9	55.9	ND	ND	ND	56.0	161.4	67.1	81.7
**IL-16**	72.6	ND	ND	ND	ND	ND	115.8	133.6	ND	124.2	88.1	157.8	82.6	95.5
**IL-17A**	103.4	22.5	ND	ND	ND	424.0	383.8	244.5	186.0	369.3	528.0	754.1	540.4	507.9
**IL-18 Bpa**	785.6	ND	371.6	103.6	56.9	879.3	912.0	886.2	560.1	879.2	850.3	904.2	795.2	703.8
**Il-19**	ND	ND	ND	ND	ND	ND	ND	ND	ND	ND	59.3	137.9	39.1	371.2
**IL-1α**	99.7	44.2	43.7	29.6	34.1	ND	ND	206.2	143.0	209.7	78.0	309.3	66.3	83.5
**IL-1β**	ND	ND	ND	ND	ND	ND	ND	ND	ND	ND	61.3	341.6	68.2	83.7
**IL-1ra**	643.1	ND	298.5	28.7	ND	495.9	489.4	696.2	573.5	659.2	609.8	557.9	449.8	481.6
**IL-2**	ND	ND	ND	ND	ND	ND	ND	92.7	151.9	458.1	220.9	317.8	164.6	127.5
**IL-22**	70.1	ND	ND	ND	ND	98.8	105.3	102.3	58.6	98.1	349.9	406.5	271.8	281.2
**IL-23**	ND	ND	ND	ND	ND	ND	ND	ND	ND	ND	47.4	129.9	48.5	68.6
**IL-24**	137.5	ND	ND	ND	ND	ND	158.0	136.1	ND	149.8	146.2	216.7	125.0	124.2
**IL-27**	110.6	ND	ND	ND	ND	ND	ND	104.2	165.9	297.2	60.3	199.1	184.6	70.4
**IL-3**	ND	ND	ND	ND	ND	ND	ND	ND	84.4	ND	38.8	158.6	54.8	118.5

	AML#3	AML#18	AML#23	HL-60	U937	AML#3 +Mo	AML#23 +Mo	M-MΦ	GM-MΦ	$R^{GM/GW}$-MΦ	$HD^{AML\#3}$-MΦ	$HD^{AML\#3}$-MΦ +GM/GW	$HD^{AML\#23}$-MΦ	$HD^{AML\#23}$-MΦ +GM/GW
IL-31	ND	ND	ND	ND	ND	ND	ND	ND	55.3	121.3	63.5	106.5	121.8	82.4
IL-32	ND	ND	ND	ND	ND	ND	ND	62.1	45.6	98.1	84.9	86.8	50.2	71.5
Il-33	ND	ND	ND	ND	ND	ND	ND	ND	ND	ND	75.2	90.8	67.1	80.5
IL-34	ND	ND	ND	ND	ND	ND	ND	ND	ND	ND	64.1	139.0	64.9	86.9
IL-4	ND	ND	ND	ND	ND	75.6	86.5	ND	ND	ND	253.6	377.7	259.1	275.5
IL-5	304.3	52.5	48.3	ND	66.9	ND	ND	302.3	266.1	355.4	15.3	23.0	12.8	11.9
IL-6	ND	ND	ND	ND	ND	75.9	113.0	114.8	172.8	418.9	252.3	974.8	885.5	912.0
IL-8	1002.8	44.7	916.5	924.7	863.5	1014.7	997.2	958.6	979.6	995.2	1028.9	1053.5	997.5	1013.7
Kallikrein 3	143.4	89.2	115.5	84.5	72.2	ND	ND	345.2	284.6	394.1	192.8	307.2	170.0	188.8
Leptin	ND	ND	ND	ND	ND	ND	ND	ND	ND	ND	96.8	91.8	47.2	58.1
LIF	ND	ND	ND	ND	ND	ND	ND	137.6	ND	84.6	48.5	114.5	42.4	43.8
Lipocalin-2	270.9	51.9	74.6	39.9	62.7	65.1	344.3	218.7	228.1	207.5	83.8	110.2	65.4	60.1
M-CSF	124.7	29.2	ND	ND	ND	269.2	ND	508.0	208.1	387.6	926.6	409.6	522.9	402.5
MIF	323.1	523.0	424.8	326.0	397.4	374.9	387.3	410.1	324.6	489.0	462.2	556.2	329.9	488.6
MMP-9	821.5	ND	788.1	822.7	108.0	838.2	816.0	887.5	815.0	928.9	1011.9	1044.3	1027.1	997.9
MPO	246.2	44.6	491.7	86.5	43.6	277.8	802.2	320.3	297.6	291.9	91.0	58.0	370.4	246.4
Osteopontin	918.1	28.8	804.0	843.6	43.8	898.1	824.8	929.8	913.6	924.3	801.4	811.9	848.7	777.3
PDGF-AA	79.5	30.5	ND	64.4	42.2	387.1	147.1	154.8	99.8	99.5	148.2	299.2	367.0	383.0
PDGF-AB/BB	ND	ND	ND	ND	ND	373.5	108.4	455.7	209.1	317.7	372.7	409.1	493.2	382.9
PECAM-1	337.6	ND	258.3	367.1	ND	837.9	685.0	762.4	563.1	832.8	537.5	749.1	506.7	599.5
PTX3	223.4	113.1	134.8	104.3	93.4	83.1	72.9	353.5	283.0	350.1	216.4	415.7	225.3	293.4
RAGE	ND	ND	ND	ND	ND	ND	ND	ND	ND	ND	64.5	69.0	56.1	85.5
RBP-4	ND	ND	ND	ND	ND	84.9	62.6	ND	ND	ND	113.0	223.3	98.8	133.1
Relaxin-2	83.5	ND	ND	ND	ND	ND	ND	112.1	94.7	176.7	55.6	97.5	71.2	98.2
Resistin	87.8	48.4	154.5	1036.1	88.8	118.6	209.1	146.2	101.9	213.7	240.4	358.9	193.6	226.1
Serpin E1	992.2	369.5	762.5	966.7	828.8	1050.5	1032.4	1019.9	1017.0	1005.1	925.8	803.0	781.1	742.2
SHBG	139.3	ND	ND	ND	ND	162.7	116.6	195.6	127.4	197.9	139.9	115.3	125.6	104.0
TFF3	ND	ND	ND	ND	ND	ND	ND	ND	ND	ND	52.1	89.4	50.4	64.5
TGF-α	142.1	ND	ND	ND	ND	108.3	ND	122.8	197.8	340.6	33.5	156.1	32.4	121.7
Thrombospondin-1	578.9	ND	179.9	39.9	ND	590.4	440.0	425.1	202.0	183.3	445.9	621.3	271.9	273.1
TIM-3	870.6	ND	116.8	55.9	ND	954.5	647.2	928.5	823.9	916.7	925.0	753.5	764.1	801.2
TNF-α	ND	ND	ND	ND	ND	212.5	220.2	110.7	574.6	587.4	54.8	804.7	80.4	201.0
uPAR	888.2	22.6	461.3	598.0	261.6	854.6	754.5	847.7	815.4	890.7	843.1	860.3	808.5	826.9
VCAM-1	556.4	ND	ND	ND	ND	230.3	290.7	690.3	79.0	220.3	67.3	87.4	68.6	131.2
VEGF	ND	934.7	ND	1064.0	780.4	ND	ND	78.1	ND	130.5	86.2	895.7	679.4	476.3
Vitamin D BP	59.4	52.0	48.0	54.8	40.4	78.4	52.0	208.0	176.2	274.2	111.4	143.3	93.5	104.0
